# Significance of Epigenetic Alteration in Cancer-Associated Fibroblasts on the Development of Carcinoma

**DOI:** 10.3390/ijms26199695

**Published:** 2025-10-05

**Authors:** Hongdong Gao, Hinano Nishikubo, Dongheng Ma, Juncheng Pan, Tomoya Sano, Daiki Imanishi, Takashi Sakuma, Canfeng Fan, Masakazu Yashiro

**Affiliations:** 1 Japan Molecular Oncology and Therapeutics, Osaka Metropolitan University Graduate School of Medicine, 1-4-3 Asahimachi, Abeno-ku, Osaka 545-8585, Japan; gaohomgdong99@gmail.com (H.G.); sn23089k@st.omu.ac.jp (H.N.); sg24231c@st.omu.ac.jp (D.M.); junchengpan0723@gmail.com (J.P.); sb24524y@st.omu.ac.jp (T.S.); sy23003h@st.omu.ac.jp (D.I.); so22500y@st.omu.ac.jp (T.S.); fancanfeng@gmail.com (C.F.); 2 Cancer Center for Translational Research, Osaka Metropolitan University Graduate School of Medicine, 1-4-3 Asahimachi, Abeno-ku, Osaka 545-8585, Japan

**Keywords:** epigenetic alteration, CAF, DNA methylation, histone acetylation, tumor microenvironment

## Abstract

Cancer-associated fibroblasts (CAFs) are a key constituent of the tumor microenvironment. CAFs may affect the development of tumor cells. The critical role of CAFs in the tumor microenvironment is linked to their epigenetic modifications, as a stable yet reversible regulation of cellular phenotypes. Current evidence indicates that their formation and function are closely linked to epigenetic mechanisms. Existing research indicates that the epigenetic alteration abnormalities are triggered by metabolic cues and stabilize the acquired phenotype of CAFs. This process is associated with transcriptional changes and patient outcomes in various tumors, providing a biological rationale and translational potential for reprogramming CAFs. Understanding of epigenetic modifications in CAFs remain insufficient, while DNA methylation in CAFs can alter CAF states through multiple pathways and thereby influence tumor progression. It is necessary to investigate the unique, identifiable epigenetic signatures of CAF. As an epigenetic reader couple histone acetylation to high-output oncogenic transcription; meanwhile, noncoding RNAs modulate CAF formation and therapeutic responses via bidirectional crosstalk between tumor cells and stroma. The interactions between different epigenetic modifications and their underlying regulatory logic may play a crucial role in developing new therapeutic strategies. This review focuses on the roles of DNA methylation, histone acetylation, and enhancer reprogramming in CAFs.

## 1. Introduction

During tumor development, the tumor microenvironment (TME) exerts a major influence on disease trajectory and therapeutic behavior [1]. The TME comprises immune cells and abundant stromal cells [2,3,4]. Stromal cells constitute the majority and include endothelial cells, mesenchymal stromal cells (MSCs), pericytes, and cancer-associated fibroblasts (CAFs) [5]. CAFs engage in bidirectional communication with tumor and immune cells, modulate the extracellular matrix (ECM), and remodel the microenvironment, thereby shaping tumor progression and therapeutic responses [6,7]. As key stromal cells within the TME, CAFs coordinate matrix deposition and remodeling together with paracrine regulation, influencing proliferation, invasion, angiogenesis, immune modulation, and treatment responses [8,9]. They display pronounced heterogeneity and plasticity. Recent reports indicated that CAF activation and maturation coincide with reprogramming of DNA methylation and histone modifications, partly driven by TGF-β, supporting epigenetic strategies to reset CAF states [10,11,12,13].

Epigenetic mechanisms—including DNA methylation, histone modifications, chromatin architecture, and non-coding RNAs—provide a stable yet reversible regulatory layer that directs gene expression programs and cellular phenotypes. Their central roles including tumor initiation, metastasis, and therapeutic response have been repeatedly demonstrated [14,15,16]. Although evidence connecting CAF biology and epigenetics is accumulating, systematic reviews focused specifically on this intersection remain scarce relative to literature on each field alone. Despite lacking uniform somatic driver mutations, CAFs undergo widespread reprogramming of DNA methylation and histone marks during activation or maturation, including loss of histone methylation and increased acetylation [17,18]. Bromodomain and extra-terminal domain (BET) proteins function as epigenetic readers that recognize histone acetylation and recruit transcriptional elongation machinery, thereby amplifying enhancer-driven gene expression [19]. Pharmacologic BET inhibition can reprogram oncogenic transcriptional networks and, in pancreatic ductal adenocarcinoma (PDAC) models, suppress CAF-associated stromal responses, highlighting therapeutic and reprogramming opportunities [20]. Population-level and clinically oriented signals are also emerging: CAFs across tumor types exhibit aberrant DNA methylation with accompanying transcriptional changes and associations with patient survival. These observations suggest utility for patient stratification and hypothesis-driven translational research across multiple disease settings [21].

Beyond canonical DNA methylation and histone modifications, recent studies implicate the tumor microenvironment (TME) in epigenetic changes within cancer-associated fibroblasts (CAFs). Key TME processes include immunometabolic reprogramming and extracellular vesicle mediated intercellular communication, both of which may modulate the CAF epigenome [22,23]. Immunometabolic reprogramming denotes metabolic shifts within immune cells that reshape outputs of metabolites and cytokines. In macrophages, a glycolysis, hypoxia, succinate and hypoxia-inducible factor 1 alpha (HIF-1α) pathway increases interleukin-1 beta (IL-1β) production. These immune mediators can influence the emergence and maintenance of distinct CAF subpopulations within tumors [24,25,26].

In pancreatic ductal adenocarcinoma (PDAC), interleukin-1 (IL-1) induces leukemia inhibitory factor (LIF) and downstream Janus kinase/signal transducer and activator of transcription (JAK/STAT) activation to generate inflammatory CAFs (iCAFs).By contrast, transforming growth factor beta (TGF-β) downregulates interleukin-1 receptor type 1 (IL1R1) and thereby promotes myofibroblastic CAFs (myCAFs). In addition, extracellular vesicles (EVs) released by CAFs carry non-coding RNAs (ncRNAs) that can influence drug responses and metastatic potential. As carriers, EVs deliver ncRNAs that target epigenetic regulators or their pathways in recipient cells. In CAFs, the same mechanism may lead to epigenetic alterations as well [26,27,28,29,30]. Here, we review links between CAFs and epigenetic regulation, synthesize key advances from recent years, and discuss translational strategies and unresolved questions.

### 1.1. DNA Methylation

DNA methylation mainly refers to the conversion of cytosine at CpG sites into 5-methylcytosine (5mC); in mammals, approximately 70–80% of CpG sites carry 5mC [31]. These CpGs often cluster in the genome to form CpG islands (CGIs); when CGIs located at gene promoters become hypermethylated, chromatin tends to adopt a closed state and gene transcription is repressed [32]. DNA methylation is catalyzed by DNA methyltransferases (DNMTs): DNMT3A/3B mediate de novo methylation, and DNMT1 recognizes hemimethylated DNA at the replication fork to perform maintenance methylation; S-adenosyl-methionine (SAM) serves as the methyl donor, ensuring faithful transmission of methylation patterns across cell divisions [33,34].

By contrast, TET1/2/3 oxidize 5mC stepwise to 5hmC to 5fC to 5caC, which can be actively removed through TDG-mediated base excision repair (BER); if DNMT1 fails to “fill in” methylation after replication, passive (replication-dependent) demethylation occurs [35,36]. In tumors, global hypomethylation coexists with focal promoter/CGI hypermethylation, a repeatedly validated epigenetic hallmark that often arises early in tumorigenesis and intensifies with progression [37]. Functional consequences include genome-wide hypomethylation is associated with genomic/chromosomal instability (with causal evidence in mouse models); promoter CGI hypermethylation can silence tumor-suppressor genes, impacting key pathways such as the cell cycle, apoptosis, DNA repair, and angiogenesis [38,39]. From an enzymology and clinical association perspective, upregulation of the DNMT family is observed in multiple cancers and correlates with poor prognosis in several settings; conversely, many tumors exhibit suppressed TET activity with widespread loss of 5hmC (which carries diagnostic/prognostic significance in, e.g., melanoma) [40,41].

### 1.2. Tumors and DNA Methylation (Table 1)

DNA methylation provides a stable and heritable epigenetic layer that can lock cell phenotypes, making it a key mechanism for establishing and maintaining pro-tumor cancer-associated fibroblast states. Aberrant methylation in CAFs is not random but is actively driven by specific signals within the tumor microenvironment. These drivers include classic mediators such as transforming growth factor beta (TGF-β), which can recruit DNA methyltransferases (DNMTs) to selected gene promoters; however, the direction of change varies by fibroblast tissue origin, indicating mechanisms that are plastic yet constrained. Metabolic reprogramming also contributes; for example, high lactate can activate ten eleven translocation dioxygenases (TETs) and induce widespread demethylation [22,42,43,44]. 

In pancreatic ductal adenocarcinoma, lactate activates TETs, increases 5-hydroxymethylcytosine (5hmC), and promotes broad demethylation that drives the transition from normal-associated fibroblasts (NAFs) to CAFs. Importantly, cross-cancer integrative analyses have repeatedly identified CAF-associated CpG changes, including cg09809672 near EDARADD, cg07134930 near HDAC4, and cg05935904 [21,22].

These observations suggest that CAF-related DNA methylation may serve as prognostic markers. The resulting methylome alterations are not merely correlative; they have direct functional consequences, such as silencing tumor suppressor genes or activating oncogenic pathways, thereby stably reprogramming fibroblasts to support tumor progression [21,42,45].

We will next introduce, by cancer type, the associations between CAF emergence and DNA methylation. Across diverse malignancies, we will examine evidence for these principles to clarify both cross-cancer methylation imprints and tumor-specific methylation patterns that record the distinctive biological influences exerted by different microenvironments.

**Figure 1:**
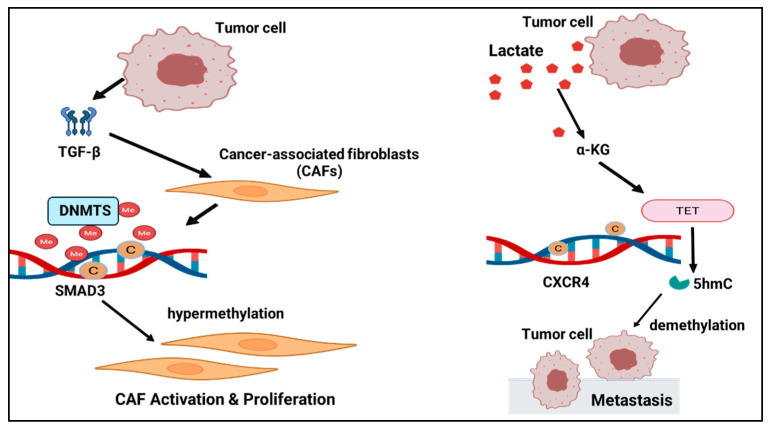
CAF methylation in various tumor microenvironments.

DNA methylation in pancreatic ductal adenocarcinoma (PDAC)—PDAC features a sclerotic stroma with fibroblasts comprising 90% of tumor mass. Tumor cells can induce DNA methylation in normal adjacent fibroblasts (NAFs), converting them into CAFs and promoting PDAC progression. Direct tumor–CAF contact induces SOCS1 promoter methylation and SOCS1 downregulation, activating STAT3 and increasing IGF-1 expression to support tumor growth [12,22,43,46]. Combined inhibition of the G9a–DNMT1–UHRF1 complex has been proposed as a therapeutic strategy, underscoring the druggability of methylation machinery within the pancreatic stroma. PDAC displays widespread, stage-linked methylome alterations that provide a framework for causal methylation–function tests at the level of CAF subsets [47,48,49]. Metabolic control of CAF methylation in PDAC—A high-lactate milieu can raise α-ketoglutarate (α-KG) and activate TET dioxygenases, producing genome-wide demethylation and 5-hydroxymethylcytosine (5hmC) accumulation in CAFs. Under high lactate, CXCR4 undergoes demethylation with substantial 5hmC gain and marked transcriptional upregulation; elevated CXCR4 increases CAF-derived chemokines and enhances tumor invasiveness [22]. In mouse PDAC models, primary tumors with a liver-metastatic propensity program distant tissues to activate more CAFs and alter methylation at metabolic genes, whereas lung-tropic tumors show weaker remote programming. These differences may underline organ-specific clinical patterns and point to DNA demethylating agents or blockade of key axes such as CXCR4 to reprogram CAFs and restrain progression (Figure 1) [22,49,50].

DNA methylation in breast cancer—The CAF methylome shows widespread epigenetic reprogramming closely tied to RUNX1 upregulation, indicating crosstalk between transcription-factor networks and methylation during CAF activation [17,51,52]. During conversion of normal fibroblasts to CAFs, RUNX1 and stromal gene markers are strongly induced; high RUNX1 expression in CAFs associates with worse prognosis in breast cancer. The RUNX1/Syndecan-1 (SDC1) axis is particularly active in invasive CAFs: RUNX1 directly activates SDC1, whose protein product contributes to extracellular-matrix remodeling and promotes tumor cell migration and metastasis. These findings mechanistically explain how RUNX1-high CAFs drive disease progression and suggest the RUNX1/SDC1 axis as a potential therapeutic target [17,53].

DNA methylation in hepatocellular carcinoma (HCC)—Joint methylome–transcriptome profiling of primary human HCC-derived CAFs shows CAF-specific CpG abnormalities tightly coupled to expression changes, with CAF-specific methylation levels correlating with patient survival. Cross-cancer integration of lung, prostate, esophageal, and gastric CAF datasets reveal shared regions of aberrant methylation, indicating transferable CAF methylation marks across tumor types [21]. Moreover, tumor-derived CCL15 promotes HCC growth by modulating N6-methyladenosine (m6A) in CAFs, highlighting RNA epigenetics as an additional regulatory layer that reinforces CAF epigenetic plasticity [54].

DNA methylation in lung cancer—Lung CAFs display a pattern of overall demethylation with focal hypermethylation; non-small-cell lung cancer (NSCLC) exhibits broad methylation abnormalities. One study reported 14,781 differentially methylated CpGs in NSCLC CAFs compared with paired normal fibroblasts (NFs) [55]. Promoter hypermethylation of SMAD3 is a prominent marker of lung CAFs [56]. TGF-β is a major upstream driver: in TGF-β-rich tumors, CAF nuclei may recruit additional DNMTs, leading to selective promoter hypermethylation of TGF-β target genes such as SMAD3, thereby locking CAFs in an activated state. Selective epigenetic suppression of SMAD3 has also been used to explain the limited efficacy of anti-fibrotic agents in lung squamous carcinoma, pointing to CAF methylation states as modifiers of treatment response. In heavily pretreated metastatic NSCLC, low-dose azacitidine combined with entinostat achieved objective and durable responses in a phase I/II study; pharmacodynamic demethylation correlated with benefit and suggested a priming effect for subsequent therapies [57]. By contrast, in PD-(L)1-experienced patients, the phase Ib/II ENCORE-601 trial of entinostat plus pembrolizumab yielded an objective response rate of approximately 9%, failed to meet its prespecified efficacy threshold, and did not assess CAF-related endpoints. In previously treated advanced NSCLC, a randomized phase II trial of oral azacitidine (CC-486) plus pembrolizumab versus placebo plus pembrolizumab did not demonstrate a significant clinical benefit. DNA methylation in CAFs contributes to lung cancer progression and may represent a therapeutic target. Current data support the feasibility of combining epigenetic agents with immune-checkpoint blockade in NSCLC; however, patient-level evidence that these regimens confer benefit through CAF-specific epigenetic remodeling is lacking. CAF epigenetics remains a putative therapeutic entry point that warrants prospective validation [58,59,60,61].

DNA methylation in oral squamous cell carcinoma (OSCC)—Fibroblast nicotinamide N-methyltransferase (NNMT) promotes angiogenesis and tumor growth through an “epigenetic reprogramming—ETS2—VEGFA” axis, emphasizing a metabolic–epigenetic interface that shapes pro-angiogenic CAF phenotypes [62]. Similar regulatory roles for CAF-NNMT have been observed in lung adenocarcinoma, suggesting broader generalizability [63,64].

DNA methylation in colorectal cancer (CRC)—CAFs in primary CRC differ from adjacent normal fibroblasts across multiple signaling pathways; for example, Wnt/β-catenin target genes are upregulated in CAFs. Wnt/β-catenin signaling can induce PKP2 (Plakophilin-2) expression in both normal colonic fibroblasts and CRC-CAFs, indicating that tumor-derived Wnt factors directly reprogram fibroblast transcriptional programs [65,66]. Genome-wide methylation analysis using the Illumina Human Methylation27 BeadChip showed systematic differences in promoter methylation and transcription between CRC CAFs and NFs, with expression–methylation coupling that supports epigenetic reprogramming as a driver of stable CAF phenotypes [65]. In sporadic, non-familial CRC, late-stage tumors show global DNA hypomethylation in stromal CAFs, using LINE-1 as a surrogate, suggesting progressive methylation erosion with disease advancement [67]. In non-familial sporadic CRC, CAFs exhibit stage-dependent global DNA hypomethylation assessed by nuclear 5-methylcytosine (5mC) immunostaining [68]. In the context of colorectal liver metastasis (CRLM), methylome profiling of directly isolated CAFs and NAFs showed that the CAF-high subset, compared with NAFs, exhibited about 2838 hypomethylated CpGs and about 1144 hypermethylated CpGs; hypomethylation was most prominent in the regulatory region of COL1A1, whereas the GJA4 region was hypermethylated. These differences may be related to CAF activation and warrant further investigation [21].

Cross-cancer integration—Analyses incorporating CAF datasets from lung, prostate, esophageal, and gastric cancers identify recurrent hypomethylation at sites such as EDARADD (cg09809672) and HDAC4 (cg07134930). These loci correlate with survival across several TCGA cohorts and may serve as methylation biomarkers of CAF burden or state, pending further validation [21].

DNA methylation in gastric and esophageal adenocarcinoma—Multi-omics studies of human CAFs first delineated DNA methylation differences between CAFs and patient-matched stromal fibroblasts [21]. Gastric CAFs harbor widespread differentially methylated sites and share labile CpGs with esophageal CAFs, supporting a common mode of CAF epigenetic reprogramming. Candidate-gene validation shows inverse methylation–expression relationships for SMAD3 and SPON2 promoters in gastric CAFs, confirmed by pyrosequencing in paired samples [69]. Independent cross-cancer meta-analysis that includes multiple gastric CAF datasets (e.g., GSE117087, GSE194259) likewise identifies consistently hypomethylated sites such as EDARADD and HDAC4 in gastric CAFs, reinforcing shared CAF epigenetic imprints. A key study further indicates that the pro-tumor capacity of gastric CAFs is largely determined by histone-mark regulation, especially endogenous loss of the repressive mark H3K27me3. In CAFs, loss of H3K27me3—rather than DNA methylation changes—concentrates at genes related to stem-cell niches, cell growth, and stromal–epithelial interactions, driving aberrant overexpression and secretion and underscoring the central role of histone-level alterations in CAF phenotypes [70].

Syntheses across malignancies indicate that the CAF methylome is a central integrator of oncogenic signals within the TME. Collectively, these data support a model in which upstream drivers, from signalling cytokines to metabolic by-products, converge to shape a durable pro-tumour epigenetic landscape in CAFs. More specifically, the TGF-β axis can upregulate or mobilise DNMTs, as illustrated by SMAD3 promoter methylation in lung fibroblasts, thereby stabilising a myofibroblastic phenotype. A lactate-rich metabolic milieu can activate TET, induce 5hmC gains and broad demethylation, and promote the NAF to CAF transition. Both inputs converge on promoter and enhancer methylation states that stabilise CAF paracrine output and matrix remodelling programmes.

This reprogramming appears in two complementary modes. First, specific hypomethylated sites, for example CpGs near EDARADD and HDAC4 including cg09809672, cg07134930, and cg05935904, recur across cancers and indicate a core pan-cancer programme for CAF activation; low methylation at these sites associates with poor prognosis. Second, tumour-specific methylation events, such as SOCS1 promoter hypermethylation in PDAC, highlight customised modifications imposed by distinct microenvironments; SOCS1 downregulation aligns with activation of the STAT3 and IGF-1 axis, consolidating pro-tumour paracrine activity.

Although these findings open routes for translation, attempts to reverse such changes in NSCLC have yielded mixed results. Early studies of low-dose azacitidine plus entinostat observed objective and durable responses. Subsequent entinostat plus pembrolizumab in ENCORE-601 among PD-(L)1–experienced NSCLC showed an objective response rate near 9 percent and did not meet primary endpoints. A randomised phase II trial of CC-486, the oral azacitidine, combined with pembrolizumab likewise did not show clear benefit. These observations suggest a need for CAF-anchored precision interventions rather than indiscriminate demethylation.

Future progress will depend on strategies that precisely target the CAF methylome while incorporating new complexity in CAF heterogeneity and functional subsets. Key gaps include scarce single-cell and spatial methylome evidence for CAF subpopulations and niches, which limits fine-grained causal chains from signal to methylation to function. Most studies remain correlational and lack causal editing of specific sites or enhancers with direct functional readouts, and time-resolved links between dosing, methylation kinetics, and functional responses are limited.

Based on these gaps, three testable hypotheses are proposed to guide research and translation. In lactate-enriched TMEs, CAFs exhibit TET-dependent enhancer demethylation with 5hmC gains that co-occur with high output of chemotactic and inflammatory genes; this can be tested using oxBS-seq with TET inhibition or genetic perturbation. Recruitment or upregulation of DNMT1 downstream of TGF-β forms a shared promoter-methylation pathway across cancers; this can be evaluated in gastric and colorectal CAFs using CUT and RUN for DNMT occupancy together with site-specific demethylation interventions. A CAF methylation signature comprising cg09809672–EDARADD, cg07134930–HDAC4, and cg05935904 can serve as a cross-cancer stratification score, with prospective validation on TCGA EPIC or 450 K arrays to determine thresholds and associations with CAF content and prognosis.

## 2. Histone Modifications in CAFs

Histone modification is a key mechanism of chromatin reprogramming. It regulates acetylation, methylation, and other marks at enhancers and promoters, ultimately rewriting the entire transcriptional program. The dynamic addition and removal of these modifications alter nucleosome packaging and transcription factor occupancy, resetting enhancer–promoter communication networks to drive cell state transitions and transcriptional reprogramming [71,72,73]. In the TME, this mechanism is directly related to the acquired phenotype of CAFs. Paired NAF/CAF samples from patients show that H3K27ac/H3K4me1 enhancer landscapes are rewritten at numerous sites, indicating that CAF formation and maintenance are accompanied by chromatin reprogramming at the enhancer level. The following sections will provide a detailed explanation of this process [13].

### 2.1. Enhancer Reprogramming and AP-1/JUN

Enhancer reprogramming is one of the hallmarks of CAF activation. Research on CAFs derived from breast cancer suggests that compared to NAFs, CAFs acquire a large number of active enhancers enriched with H3K27ac/H3K4me1. AP-1, particularly JUN, is enriched at these sites. JUN activation is necessary for the establishment and maintenance of CAF-specific enhancers and can drive pro-tumorigenic transcriptional programs. Gain- and loss-of-function studies show that activated JUN is necessary and sufficient to remodel enhancers and maintain CAF-specific enhancer activity, promoting tumour invasion in a non-cell-autonomous manner [13].

### 2.2. BET Inhibition Remodels Tumor Stroma/CAFs

BET proteins (BRD2/3/4) contain bromodomains that specifically recognize acetylated lysines on chromatin. BRD4 binds to acetylated H3K27ac, and upon binding to these acetylated enhancers or promoters, it recruits and activates the P-TEFb complex (CDK9/Cyclin T). P-TEFb then phosphorylates RNA polymerase II and its associated factors, releasing paused polymerases and promoting transcriptional elongation of target genes. Through this action, BRD4 bridges active histone marks and robust transcription, effectively linking enhancer acetylation with high-level gene expression. In cancer-associated stroma, hyper-acetylated “super-enhancers” can thus sustainably drive oncogenic transcriptional networks. Notably, pharmacological inhibition of the BET bromodomain (e.g., with JQ1) can prevent BRD4 from binding to these acetylated enhancers, thereby selectively suppressing SE-dependent oncogenic and inflammatory gene expression. In other words, BET inhibitors can weaken the abnormal pro-tumor transcriptional programs maintained by CAFs and tumor cells [73,74,75,76]. In pancreatic cancer patient-derived xenografts (PDX) and primary pancreatic stellate cells/CAFs, JQ1 can reduce collagen I, diminish desmoplasia, and inhibit tumor growth. These effects are not solely dependent on the sensitivity of tumor cells to JQ1 but rather rely on suppressing the pro-tumor activity and fibrotic program of CAFs. The mechanism involves AP-1/JUN facilitating the formation of new enhancers marked by H3K27ac/H3K4me1. These acetylated marks are “read” by BRD4, which then recruits P-TEFb to amplify a high-output network of pro-inflammatory and pro-stromal genes, maintaining CAF activation and desmoplasia. Interrupting this step with BET inhibitors like JQ1 can lead to CAF deactivation and stromal softening, resulting in tumor suppression in vivo. Evidence from animal models and primary CAFs/PSCs shows that BET inhibitors can reduce fibrosis and inhibit the pro-tumor activity of CAFs; however, their clinical translatability requires further investigation [20,77,78]. In CAF-rich settings, stromal cues can activate a JAK2–BRD4 axis that attenuates tumour-only BET inhibition; accordingly, combining tumour-directed agents with stromal epigenetic modulators and tracking pJAK2/pBRD4 together with EV-ncRNA signatures as pharmacodynamic readouts is rational [79].

### 2.3. Effects of HDACs

Histone deacetylases (HDACs) directly shape the functional state of CAFs and re-encode the tumor immune microenvironment. In the context of the collagen-dense stroma of PDAC, broad-spectrum HDAC inhibition has been shown to induce an AP-1-related inflammatory, tumor-supportive secretory phenotype in CAFs, highlighting the risks of non-selective blockade. In contrast, selective or targeted HDAC strategies show potential for beneficial stromal remodeling [80,81]. HDACs typically remove acetyl groups, leading to chromatin compaction and transcriptional repression. Paradoxically, in some contexts, broad-spectrum HDAC inhibitors can produce unintended pro-tumor effects in the stroma. For example, in PDAC models, application of pan-HDAC inhibitors can induce an inflammatory, tumor-supportive phenotype in CAFs, characterized by elevated secretion of cytokines and chemokines that enhance cancer cell invasiveness. Conversely, selective or targeted HDAC approaches show potential for beneficial stromal remodeling. In PDAC mouse models, the Class I HDAC inhibitor entinostat can reduce CAF activation and fibrosis, inhibit tumor progression, and induce the emergence of a lipogenic progenitor-like fibroblast subpopulation. This suggests that the CAF lineage is plastic and pharmacologically reprogrammable [82].

On the other hand, certain HDAC subtypes play a critical role in maintaining the immunosuppressive and pro-tumor functions of CAFs. In breast cancer, HDAC6 is often upregulated in CAFs and influences the microenvironment through the STAT3–COX-2 pathway. Inhibiting HDAC6 can reduce the recruitment of immunosuppressive cells (Tregs/MDSCs), enhance T cell activity, and slow tumor growth. High stromal HDAC6/p-STAT3/COX-2 levels are associated with poor prognosis. Therefore, selective HDAC inhibition (e.g., targeting HDAC6) may reprogram CAFs to a more immune-supportive and less pro-tumor state. In contrast, non-selective HDAC blockade might activate pro-tumor inflammatory programs in fibroblasts. This demonstrates that the epigenetic regulation of CAFs is bidirectional, with its effects depending on the targeted chromatin regulator and the context. This indicates that HDAC interventions require strict selectivity and careful consideration of dose and timing [83].

The combination of BET and HDAC inhibitors has shown synergistic effects in various tumor models (e.g., JQ1 + HDACi in PDAC, medulloblastoma, and gastric cancer), providing a rationale for combination therapies in tumors with high CAF content. However, direct evidence for this combination specifically targeting CAFs is still lacking. Furthermore, HDAC inhibition carries context-dependent risks in the presence of CAFs. Therefore, it is advisable to use a combination of selective HDACi and BETi, with preclinical validation focusing on collagen/αSMA levels, CAF signatures, and immune infiltration as primary pharmacodynamic readouts. A working model is that, during the NAF-to-CAF transition, TET-linked demethylation precedes enhancer redistribution and is accompanied by a context-dependent reset of H3K27me3; this sequence is testable across tumour types [13,22,83,84,85,86].

## 3. Non-Coding RNAs (miRNA/lncRNA/circRNA)

It should be noted that non-coding RNAs (ncRNAs) such as microRNAs (miRNAs), long non-coding RNAs (lncRNAs), and circular RNAs (circRNAs) are not considered epigenetic modifications in the narrow sense, unlike DNA methylation and histone modifications. However, during CAF formation and maintenance, these two regulatory layers are not independent [87,88]. Non-coding RNAs indirectly reshape chromatin accessibility and transcriptional programs by regulating key signaling axes and transcription factor activities. Conversely, epigenetic modifications can alter the transcription and processing of ncRNA genes, forming a bidirectional feedback loop.

Extracellular vesicles (EVs) are lipid bilayer enclosed membranous particles released by cells that carry RNA, proteins and lipids, and mediate short- and long-range intercellular communication. In many tumour microenvironments (TMEs), EV transported microRNAs (miRNAs) and long non-coding RNAs (lncRNAs) directly regulate epigenetic pathways in recipient cells [30,89,90].

EV-transported miRNAs, lncRNAs and circular RNAs (circRNAs) can act on the epigenetic machinery of recipient cells. For example, in medulloblastoma, exosomal miR-101-3p from plasma and from monocyte- and macrophage-derived sources enters tumour cells and concurrently targets FOXP4 and EZH2, thereby suppressing tumour development. CAF-derived exosomal miR-29b directly suppresses DNMT3B expression and upregulates MTSS1, which inhibits the growth and invasion of HCC cells and modulates the expression of endothelial-to-mesenchymal transition (EMT) markers. In gastric cancer, CAF-derived exosomal miR-522 suppresses ALOX15 and lipid peroxidation, thereby limiting ferroptosis and promoting chemoresistance—illustrating how EV-miRNAs can shape epigenetic/oxidative programmes in recipient tumour cells [30,90,91,92,93].

In pancreatic ductal adenocarcinoma, CAF exosomal miR-421 downregulates the deacetylase SIRT3, leading to increased H3K9 acetylation and upregulation of HIF-1α, which promotes glycolysis and EMT. Taken together, EV-mediated transfer of ncRNAs is closely linked to CAF epigenetics and to metabolic reprogramming within the TME [94,95].

Based on this, the following discussion will explore how ncRNAs and epigenetic regulation mechanistically intertwine and converge within the framework of “non-coding RNA–epigenetic regulation–CAF phenotype” [96,97].

### 3.1. miRNA (miR-21) and CAFs

Multiple miRNAs directly regulate CAF activation and paracrine output. miR-21 repeatedly emerges as a driver of CAF activation that amplifies positive feedback within the TGF-β pathway [98]. TGF-β signaling induces miR-21 in fibroblasts; miR-21 suppresses SMAD7, relieving negative feedback on TGF-β/SMAD2/3, thereby enhancing fibroblast activation and fibrotic responses [99,100]. In PDAC, miR-21 is highly expressed in CAFs, promoting activation and resistance to gemcitabine. Elevated miR-21 correlates with increased CAF abundance and poor clinical response to gemcitabine [101]. In lung cancer, TGF-β1 secreted by lung adenocarcinoma cells induces miR-21 overexpression in normal lung fibroblasts, triggering CAF-like phenotypes with increased α-SMA and migration. Resulting CAFs, enriched for miR-21, secrete pro-tumor mediators such as calumenin, further promoting cancer-cell proliferation and migration [102,103]. Forced miR-21 expression or SMAD7 knockdown can convert normal fibroblasts into CAF-like states without exogenous TGF-β [99].

### 3.2. lncRNA and CAFs

Factors secreted by CAFs can induce changes in the expression of specific lncRNAs within tumor cells. For example, CAF-secreted TGF-β1 upregulates the long non-coding RNA HOTAIR in tumor cells, thereby promoting epithelial–mesenchymal transition (EMT) and metastasis. This reflects the epigenetic regulation of tumor cells by CAFs. Current evidence primarily supports the model of “CAFs inducing HOTAIR in tumor cells” rather than HOTAIR expression within CAFs themselves. In breast cancer, CAF-derived TGF-β1 drives HOTAIR transcription; conditioned medium from CAFs elevates HOTAIR and promotes EMT, whereas TGF-β1 inhibition attenuates this activation. Mechanistically, TGF-β-activated SMAD2/3/4 binds the HOTAIR promoter to increase transcription. In orthotopic models, HOTAIR knockdown blocks CAF-induced tumor growth and lung metastasis, linking the TGF-β1/HOTAIR axis to invasive behavior [104]. In PDAC, a platinum-resistant CAF subset (CAF^R) secretes high interleukin-8 (IL-8), which activates NF-κB in tumor cells and induces the lncRNA UPK1A-AS1.UPK1A-AS1 enhances oxaliplatin resistance by binding Ku70/Ku80 and promoting non-homologous end-joining repair of DNA double-strand breaks. Blocking the IL-8/NF-κB/UPK1A-AS1 axis restores platinum sensitivity; clinically, high UPK1A-AS1 associates with poor chemotherapy response and shorter progression-free survival [105].

In colorectal cancer (CRC), the lncRNA CCAL is highly expressed in CAFs and transferred to tumor cells via exosomes. CAF-derived CCAL interacts with HuR, enhancing β-catenin transcription and stability, suppressing apoptosis, promoting stem-like traits, and conferring resistance to 5-fluorouracil and oxaliplatin. Silencing CCAL in CAFs improves chemotherapy efficacy in mouse models, implicating CCAL in therapy resistance [106].

### 3.3. circRNA and CAFs

Exosomal circular RNAs (circRNAs) derived from CAFs are involved in regulating tumor proliferation, immune evasion, and treatment resistance. For example, certain circRNAs, such as circ_0067557 and circEIF3K, are enriched in CAF-secreted exosomes. They act as miRNA sponges or bind to specific proteins to regulate downstream signaling pathways, thereby affecting the biological behavior of cancer cells [107,108]. In colorectal cancer, circ_0067557 is significantly upregulated in exosomes secreted by CAFs compared to those from normal fibroblasts. CAF-derived circ_0067557 can be transferred to colorectal cancer cells, where it increases the expression of the oncogenes LIN28A/B. This enhances the proliferation, migration, and invasion of tumor cells and reduces their sensitivity to chemotherapy drugs like 5-FU. Mechanistic studies show that circ_0067557 can directly bind to the stem cell-associated protein LIN28, thereby affecting downstream oncogenic pathways. Knocking down circ_0067557 reduces LIN28 levels in cancer cells, inhibiting the malignant progression of colorectal cancer and partially restoring chemotherapy response. circEIF3K is a typical exosomal circRNA secreted by CAFs under hypoxic conditions. Hypoxia stimulates CAFs to release more exosomes carrying circEIF3K. Knocking down circEIF3K in CAFs significantly weakens the proliferation and invasion of colorectal cancer cells and their in vitro tube formation ability under hypoxia [108]. Further mechanistic analysis revealed that circEIF3K acts as a competitive endogenous RNA sponge for the tumor suppressor miR-214, thereby lifting the inhibition on the immune checkpoint molecule PD-L1. This promotes the growth, progression, and immune evasion of colorectal tumors. In in vivo models, knocking down circEIF3K in CAFs inhibited tumor growth and reduced PD-L1 levels in tumors, supporting its role in tumor immune evasion and progression [109].

## 4. Epigenetic Features of CAF Subsets

Cancer-associated fibroblasts (CAFs) are markedly heterogeneous. Single-cell studies consistently define at least myofibroblastic CAFs (myCAFs), inflammatory CAFs (iCAFs), and antigen-presenting CAFs (apCAFs). These subsets show clear differences in molecular programmes and functions. Single-cell studies establish myCAF, iCAF and MHC-II^+^ apCAF subsets, with apCAFs validated in PDAC and spatially resolved as mesothelial-like (tumour-proximal) and fibrocyte-like (TLS-proximal) niches [12,26,110].

myCAFs are characterised by high ACTA2 (α-SMA) expression and abundant extracellular matrix (ECM) deposition, including collagen, fibronectin, and selected MMPs. They localise near tumour nests or invasive fronts and form dense stroma that can both impede drug penetration and exert physical restraint. Upstream transforming growth factor beta (TGF-β) is a core driver; ligand–receptor binding triggers SMAD2 and SMAD3 nuclear entry to coordinate transcriptional networks and epigenetic reprogramming [12,111,112,113].

Analogous to myofibrosis, multiple studies indicate enhancer remodelling during myCAF formation, with gains of H3K27ac and H3K4me1 at de novo or strengthened enhancers. These active marks are read by BET proteins such as BRD4, which recruit the transcriptional machinery and amplify ECM and contractile gene programmes [13,114].

Paracrine factors and metabolites arising from intrinsic epigenetic imbalance in tumour cells can reshape CAF chromatin accessibility and transcription, stabilising a metabolically supportive myCAF state with open ECM and contractile loci, as shown by multi-omics in pancreatic ductal adenocarcinoma (PDAC) models [115,116].

iCAFs display a strong secretory phenotype and often express high levels of IL-6, CXCL1, and LIF. Their induction reflects inflammatory cues from tumour and immune cells; IL-1 is a key upstream signal, with the LIF–JAK–STAT3 axis supporting autocrine amplification, and NF-κB and STAT3 cooperating at many loci [26,117].

Mechanistically, JAK activation drives phosphorylated STAT3 dimerisation and nuclear entry, where it recruits acetyltransferases such as p300 and CBP to inflammatory genes. Local chromatin acetylation and accessibility increase, which drives transcription of the secretome. A 2025 cross-cancer spatial atlas further shows iCAFs forming stable cellular-neighbourhood networks with immune cells in defined niches. This suggests conserved coupling between inflammatory epigenetic programmes and tissue spatial structure; functionally, high IL-6 and STAT3 activity associates with immunosuppression and therapeutic resistance [118,119,120,121].

apCAFs upregulate major histocompatibility complex class II genes, including HLA-DR and CD74, and can interact with CD4^+^ T cells. Their immune effects are context-dependent; in gastric cancer, MHC-II-high apCAFs neighbour tertiary lymphoid structures (TLSs) and foster T-cell effector function, and higher pre-treatment apCAF abundance correlates with better immunotherapy responses.

In PDAC, apCAFs can arise from mesothelial cells and induce regulatory T cells, indicating a tolerogenic tendency [110,122,123].

Integrated single-cell and spatial analyses suggest two lineages or niches; mesothelial-like apCAFs lie near tumour nests, whereas fibroblast-like apCAFs lie closer to TLSs, offering a spatial–epigenetic explanation for pro- or anti-immune differences. In lung cancer treated with EGFR tyrosine kinase inhibitors (EGFR TKIs), CTHRC1-positive CAFs accumulate in resistant tumours and engage CTHRC1 to glycolysis to H3K18la positive feedback that drives TKI resistance; perturbing this loop restores sensitivity. Moreover, in gastric cancer, CAF-derived lysyl oxidase (LOX) can elevate tumour-cell lactate via a TGF-β→IGF-1–driven glycolytic programme; together with reports that H3K18la accumulates at the PD-L1 promoter, these data suggest a lactylation–PD-L1 axis by which CAF–tumour crosstalk may blunt PD-(L)1 blockade. In gastric cancer, translational studies show Kla-dependent suppression of NCAPG ubiquitination by CAFs, which promotes immune evasion and reinforces a causal chain from lactate to histone lactylation to immunosuppression or resistance [124,125,126].

## 5. Spatial Epigenomics in CAFs

Single-cell omics is transformative, yet tissue dissociation erases the crucial dimension of spatial context, as repeatedly noted by methodological work and reviews. Within the tumour microenvironment (TME), cell function is shaped by immediate neighbours and by position within structural and metabolic niches [127,128].

Large-scale spatial omics also links cellular neighbourhoods with immune phenotypes and clinical outcomes. CAFs adjacent to invasive fronts receive signals distinct from those in immune-rich TLS neighbourhoods, perivascular tracks, and perineural regions, and likely display different regulatory, and still under-characterised, epigenetic states. To explain CAF heterogeneity and plasticity, their epigenomes must be studied in situ. The emerging field of spatial epigenomics provides appropriate tools and promises an added layer for decoding TME complexity [121,129,130].

Spatial organisation within tumours is structured rather than random, forming a highly organised ecosystem. Cross-cancer integration shows that CAF subsets occupy specific niches and neighbourhoods. myCAF and ECM programmes concentrate at tumour–stroma borders and associate with immune exclusion, whereas iCAFs are more dispersed and often co-localise with immune infiltration. apCAFs enrich near TLSs and associate with active immune responses and better responses to immunotherapy [121,122,128,129].

These CAF neighbourhoods predict local immune phenotypes and survival across cancers, supporting a strong subset–space–function coupling. Further spatially resolved studies suggest two apCAF lineages or niches; mesothelial-like apCAFs lie closer to tumour nests, while fibroblast-like or fibromyocyte-like apCAFs lie nearer TLSs. Both groups can upregulate SPP1, which relates to primary-site formation and peritoneal metastasis, offering a spatial–epigenetic framework for pro- and anti-immune variation. In PDAC, apCAFs can derive from mesothelial cells and induce regulatory T cells, underscoring context dependence. Such spatial segregation is functionally important because it sets local gradients of signals such as TGF-β and IL-1 and metabolites such as lactate and glutamine [123,131,132,133].

These gradients sculpt CAF transcriptional and epigenetic programmes; the lactate to histone lactylation axis has been implicated in immunosuppression, therapeutic resistance, and CAF–tumour interactions, including the CTHRC1-positive CAF to H3K18la feedback under EGFR TKI exposure. From a technical standpoint, cross-platform integration of spatial transcriptomics and proteomics using systems such as CosMx and MERscope resolves conserved spatial CAF subsets and neighbourhood networks across cancers and can predict immune phenotypes and outcomes. Spatial ATAC-seq and spatial FFPE-ATAC-seq now map chromatin accessibility in situ and apply to clinical FFPE archival samples, showing good agreement with single-cell ATAC-seq. Given its compatibility with archival material, spatial FFPE-ATAC should be leveraged both to retrospectively validate subset-specific enhancer/accessibility maps and as a prospective follow-up readout in trials that modulate CAF states [121,124,127,131,134,135,136].

These advances enable direct observation of CAF chromatin states in true spatial coordinates and provide a basis for subset-specific, locus-level epigenomic maps with reproducible clinical validation [137].

## 6. Conclusions

CAFs are one of the most important stromal cells in the TME, performing regulatory functions in matrix deposition, remodeling, and paracrine signaling. They broadly affect the growth, invasion, angiogenesis, immune regulation, and therapeutic response of tumor cells, exerting a key influence in these aspects. CAF subsets are guided by antagonistic cytokine axes and resolve into distinct chromatin programmes; interleukin 1 (IL-1) to leukaemia inhibitory factor (LIF) to JAK/STAT3 directs inflammatory CAFs (iCAFs). Transforming growth factor beta (TGF-β) suppresses interleukin-1 receptor type 1 (IL-1R1) and biases myofibroblastic CAFs (myCAFs).

In pancreatic ductal adenocarcinoma (PDAC), antigen-presenting CAFs (apCAFs) demonstrably arise from mesothelial cells and can induce regulatory T cells (Tregs). Spatial analyses show that apCAFs can accumulate around tertiary lymphoid structures (TLSs) and associate with immune activation and therapeutic response. We therefore propose anchoring these state differences with subset specific enhancer and chromatin accessibility maps, integrated with spatial neighbourhoods including TLSs, invasive fronts, and perineural regions. This framework may explain why identically named subsets display divergent immune effects across tumour types and anatomical sites. The critical role of CAFs in the tumor microenvironment is closely linked to their reversible epigenetic reprogramming. Epigenetic modifications, as a stable yet reversible regulatory mechanism, directly determine gene expression profiles and cellular phenotypes. They have been repeatedly confirmed to hold a central and druggable position in tumorigenesis, metastasis, and drug response.

Across multiple cancers, a high-lactate niche co-constructed by tumours and CAFs reshapes chromatin readouts in CAFs as well as tumour cell fate. On one hand, in pancreatic ductal adenocarcinoma (PDAC), the lactate to alpha-ketoglutarate (α-KG) to ten-eleven translocation (TET) to 5-hydroxymethylcytosine (5hmC) axis links metabolism directly to DNA demethylation. On the other hand, lactate-induced histone lactylation (H3K18la) forms an amplifiable positive feedback loop across tumour and stroma that upregulates programmed death ligand 1 (PD-L1) in tumour cells and consolidates CAF programming and immunosuppressive phenotypes.

Together, these findings support metabolism to epigenetic writing and reading to cell state as a foundational mechanism in the TME that should align with therapeutic stratification and pharmacodynamic monitoring. Extracellular vesicle (EV)-borne non-coding RNAs (ncRNAs) convey signals and directly engage epigenetic targets.

CAF exosomal miR-29b suppresses DNA methyltransferase 3B (DNMT3B) in recipient tumour cells, relieving methylation repression and altering transcriptional profiles. In endometrial and bladder models, CAF EV miR-148b is also linked to DNA methyltransferase 1 (DNMT1) regulation, with context-dependent directionality, indicating that CAF-to-tumour communication can sculpt recipient chromatin via an ncRNA-to-epigenetic enzyme axis. In light of the proposal that EVs serve as a window for drug response and resistance monitoring, EV markers such as miR-29b and miR-148b associated with the DNMT axis may be incorporated into paired clinical sampling and follow-up.

Existing research indicates that DNA methylation abnormalities triggered by TGF-β and metabolic cues, an imbalance of repressive and active histone marks, and reprogramming at the enhancer level collectively shape and stabilize the acquired phenotype of CAFs. This process is associated with transcriptional changes and patient outcomes in various tumors, providing a biological rationale and translational potential for reprogramming CAFs instead of eliminating them. BET proteins, as epigenetic readers, link acetylation marks to high-output transcription. In pancreatic cancer models, BET inhibitors can alleviate desmoplasia and weaken the pro-tumor program of CAFs. The effect of HDACs is context-dependent; broad-spectrum inhibition risks pushing CAFs towards an inflammatory and pro-tumor secretory phenotype, while selective approaches hold greater potential for resetting them. In some contexts, demethylation strategies or blockades of key pathways, such as CXCR4, may interrupt the positive feedback between CAFs and tumor cells.

Therapeutic tolerance mediated by cancer-associated fibroblasts (CAFs) relies on reversible epigenetic programs. Key routes include a non-coding RNA (ncRNA) and extracellular vesicle (EV) axis, metabolism-driven histone modifications, and druggable chromatin regulators. These routes shape adaptive tolerance to chemotherapy and targeted agents, and they reset the ceiling of responses to immunotherapy. In gastric cancer, CAFs transfer exosomal miR-522 that targets ALOX15, suppresses lipid peroxidation and ferroptosis, and induces acquired chemoresistance. In pancreatic ductal adenocarcinoma, platinum-resistant patient-derived CAFs use an interleukin-8 and NF-κB axis to induce tumour cell lncRNA UPK1A-AS1, enhance double-strand break repair, and confer oxaliplatin resistance; disrupting this axis reverses tolerance. In colorectal cancer, CAF exosomal lncRNA CCAL binds HuR to stabilise β-catenin mRNA, inhibit apoptosis, and cause resistance to 5-fluorouracil and oxaliplatin.

Together, these findings indicate that CAFs establish tolerant phenotypes in tumour cells through transmissible epigenetic cargo. In EGFR-mutant lung cancer, lactate induces H3K18 lactylation in CAFs, upregulates CTHRC1, and creates CTHRC1-to-glycolysis-to-H3K18la positive feedback that drives resistance to EGFR tyrosine kinase inhibitors; CTHRC1-positive CAFs emerge as predictive and interventional targets. This work connects metabolites to histone lactylation to CAF programming and directly to adaptive resistance to targeted therapy, supporting combined metabolism and epigenetics interventions. Epigenetic constraints also shape the upper bound of immunotherapy response. In gastric cancer, CAF-secreted lysyl oxidase (LOX) increases tumour cell lactate through a TGFβ-to-IGF1-to-glycolysis pathway; H3K18la accumulates at the PD-L1 promoter and elevates its transcription, suggesting that CAFs can blunt PD-1 or PD-L1 blockade through a lactylation-to-PD-L1 axis.

Recent work shows that histone lactylation in CAFs also promotes immune evasion by suppressing ubiquitination of NCAPG, further highlighting lactylation as a key node in CAF-mediated immune tolerance. Given the risks of pan-histone deacetylase inhibitors and progress with selective agents, precise targeting of stromal histone deacetylases may reprogramme the tumour microenvironment.

These considerations support combining tumour-directed agents with stromal epigenetic modulators as a future therapeutic strategy.

## 7. Future Perspectives

Based on the findings of this study, it is hypothesized that differentially methylated CpG sites and enhancer features that are reproducible across cancer types could serve as a basis for patient stratification and as pharmacodynamic readouts.

However, their robustness and transferability require validation in multiple cohorts and prospective frameworks. The pro-tumorigenic function of cancer-associated fibroblasts (CAFs) is not determined by irreversible genetic mutations but by an epigenetic program orchestrated by DNA methylation, histone modifications, and non-coding RNA networks. This program is, in principle, pharmacologically reversible. This understanding broadens the potential for cancer treatment. Future effective cancer therapies may need to target both the cancer cells and their stroma, with the key possibly residing in CAF-related epigenetic modifications [13,17,70,82,138].

Nevertheless, this promising prospect currently faces a significant evidentiary gap. Pan-cancer epigenetic analyses, such as The Cancer Genome Atlas (TCGA) project, primarily use data from bulk tumor tissues containing various cell types, meaning their conclusions largely reflect the general characteristics of tumor cells.

Concurrently, while in-depth studies on purified CAF epigenomes have yielded valuable results, they are mostly confined to specific cancer types like breast or lung cancer. Therefore, whether a core, conserved CAF activation epigenetic program exists across multiple cancers remains a critical unanswered scientific question. Future research should prioritize filling this key knowledge gap through systematic, multi-cancer CAF cohorts.

Recent datasets indicate that CAFs exhibit reproducible DNA methylation aberrations and enhancer reprogramming features that recur across tumor types; these could be developed into CAF epigenetic signature loci for patient stratification and therapeutic monitoring. We foresee two future directions. A fixed set of consistently, differentially methylated regions reproducible across multiple cancer types could be used for patient stratification, prediction of treatment efficacy, and as pharmacodynamic readouts. The establishment of such a loci set would enable cross-cancer transferability and cross-platform measurability. This suggests a future direction for clinical stratification and efficacy monitoring by incorporating CAF-related epigenetic biomarkers. Examples include the spatial enrichment of CTHRC1+ (H3K18la-high) CAFs and the abundance of extracellular vesicle (EV)-ncRNAs (miR-522/UPK1A-AS1/CCAL) as measurable readouts for predicting responses to chemotherapy, targeted therapy, or immunotherapy. Furthermore, FFPE-ATAC and spatial epigenomics now allow for the in situ analysis of chromatin accessibility in archival samples, providing a technical path for the retrospective validation of these markers and the localization of resistance hotspots. We believe that CAF-mediated therapeutic resistance is not caused by a single pathway. Instead, it is the coupled result of a plastic epigenetic network acting on multiple axes, including “EV-ncRNA to ferroptosis/repair,” “lactate-lactylation to immune checkpoint/CAF programming,” and “chromatin writer/reader reprogramming via HDAC/BET.” Future research should prioritize the spatial characterization of resistance-related CAF programs in patient-derived samples. Additionally, clinical trials should test the biological and imaging/ctDNA biomarker endpoints of combining chemotherapy/targeted/immune therapies with CAF-targeting epigenetic drugs, such as Class I HDAC inhibitors. A CAF-rich tumor microenvironment (TME) can induce JAK2-BRD4 signaling, thereby attenuating BET inhibition, which implies that tumor-only epigenetic therapies may face stroma-driven escape. Future trials should preemptively combine tumor-directed drugs with stromal epigenetic modulators and track resistance biomarkers such as pJAK2/pBRD4 and EV-ncRNAs.

The spatial distribution of CAFs within the TME, such as their proximity to cancer nests, nerves, and blood vessels, may influence their epigenetic state and function, aiding further exploration in this field. FFPE-ATAC can now analyze chromatin accessibility in situ from archival samples, which overcomes the loss of spatial information from tissue dissociation. Integration with multiplexed transcriptomics/proteomics (e.g., CosMx, MERFISH/MERscope) can define resistance/response-associated microdomains, such as CTHRC1+/H3K18la-high CAFs and apCAF-TLS, in their native coordinates and link them to clinical outcomes and recurrence risk. We recommend its use as a validation tool for retrospective cohorts and a follow-up readout in prospective studies. When combined with EV-ncRNAs (including the DNMT axis) and immunometabolic indicators, it can form a tripartite “spatial-liquid-functional” evidence chain. Research on CAF subtypes (myCAF, iCAF, apCAF, etc.) and their specific epigenetic modifications is still exploratory. It is necessary to determine whether these functionally distinct subtypes correspond to unique, identifiable epigenetic modifications. A core knowledge gap exists: while we can classify CAF subtypes based on single-cell transcriptomics, it is unknown whether these functionally diverse transcriptional states are anchored and maintained by stable, unique, and identifiable underlying epigenetic programs, such as DNA methylation patterns and enhancer landscapes. Currently, studies directly comparing the panoramic epigenetic maps of different CAF subtypes are almost nonexistent. Answering this question is crucial for understanding the stability and plasticity of CAF heterogeneity and for developing subtype-specific epigenetic drugs.

Given the antagonistic functions of iCAFs/myCAFs (IL-1/JAK-STAT3 versus TGF-β) and the mesenchymal origin and immunomodulatory roles of apCAFs, spatially resolved enhancer/accessibility maps are needed to align CAF subtype programs with their microniches (e.g., tertiary lymphoid structure borders, invasive fronts, perivascular/perineural regions) and clinical responses. New apCAF niche atlases and datasets on perineural invasion-associated CAFs advance this agenda. During the transition from normal fibroblasts (NAFs) to CAFs, whole-genome bisulfite sequencing of breast CAFs shows large-scale demethylation and the emergence of a RUNX1-associated program. Multi-omics data suggest CAF activation is accompanied by H3K27ac enhancer reprogramming, and studies in gastrointestinal tumors report that H3K27me3 loss is linked to pro-tumorigenic CAF programs. It can be inferred that the NAF-to-CAF transition may universally involve a sequence of demethylation, followed by enhancer redistribution, and an accompanying H3K27me3 reset. Understanding the potential crosstalk among different epigenetic modifications and their intrinsic regulatory logic could be pivotal in developing new therapeutic strategies. It is also necessary to address whether resistance may develop to epigenetic reprogramming therapies designed to modulate CAFs.

## Figures and Tables

**Table 1:** CAF methylation in various tumor microenvironments.

Cancer Type	Methylation Gene in CAF	Direction
Pancreatic cancer	*SOCS1*; STAT3/IGF-1 axis	Hyper
Breast cancer	*RUNX1/Syndecan-1* (*SDC1*)	Hyper
Hepatocellular carcinoma	*CCL15*	Hyper
Lung cancer	*SMAD3*; TGF-β-driven	Mixed/hyper (SMAD3)
Oral cancer		
Colorectal cancer	*LINE-1*, *COL1A1*, *GJA4*	Global hypo
Gastric cancer and Esophageal cancer	*SMAD3*, *SPON2*; H3K27me3 loss	Promoter hyper
